# Exciton transport in atomically thin semiconductors

**DOI:** 10.1038/s41467-023-38556-9

**Published:** 2023-06-10

**Authors:** Ermin Malic, Raül Perea-Causin, Roberto Rosati, Daniel Erkensten, Samuel Brem

**Affiliations:** 1 Department of Physics, Philipps-Universität Marburg, 35032 Marburg, Germany; 2 Department of Physics, Chalmers University of Technology, 412 96 Gothenburg, Sweden

**Keywords:** Electronic properties and materials, Two-dimensional materials

## Abstract

In this Comment, the authors discuss the current status, the challenges, and potential technological impact of exciton transport in transition metal dichalcogenide (TMD) monolayers, lateral and vertical heterostructures as well as moiré excitons in twisted TMD heterostacks.

## Teaser

Atomically thin semiconductors have been in the center of one of the most active research fields. Here, we discuss the main challenges in exciton transport that is crucial for nanoelectronics. We focus on transport phenomena in monolayers, lateral heterostructures, and twisted heterostacks of transition metal dichalcogenides.

**Fig. 1:**
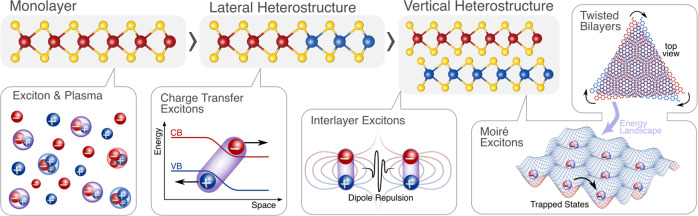
Schematic illustrating different TMD material systems and the corresponding Coulomb complexes governing the transport behavior. This includes (i) TMD monolayers with coexisting bound excitons and the electron–hole plasma at elevated densities, (ii) lateral heterostructures with spatially separated charge transfer excitons, (iii) vertical heterostructures with dipolar interlayer excitons, and (iv) twisted TMD bilayers with moiré excitons.

Transition metal dichalcogenides (TMD) have emerged as an ideal material platform for exploring exciton transport phenomena. The reduced dimensionality and weak screening lead to a remarkably strong Coulomb interaction, giving rise to the formation of tightly bound excitons that are stable even at room temperature. TMDs host a rich exciton landscape, including bright, momentum-dark, and spin-dark excitons, which govern the spatial propagation. A key advantage of TMDs is that they can be laterally stitched and vertically stacked to form heterostructures with tailored properties. This gives rise to a new type of spatially separated excitons with large in-plane or out-of-plane dipole moments that can be tuned via electric fields, cf. Fig. 1. Furthermore, TMD monolayers in a heterostack can be twisted resulting in a long-range moiré pattern that can act as a trap for excitons strongly impeding their propagation. While there has been a significant advance in the understanding of linear and non-linear exciton transport phenomena in TMD monolayers, there is still relatively little knowledge about the propagation of interlayer, charge transfer, and moiré excitons. Here, we discuss the current status, the challenges, and potential technological impact of exciton transport in TMD monolayers, lateral and vertical heterostructures as well as moiré excitons in twisted TMD heterostacks.

## Exciton transport in TMD monolayers

Due to their easy preparation, TMD monolayers offer a simple and accessible platform to investigate the underlying many-particle mechanisms behind exciton transport. Here, the dielectric environment plays a crucial role: While TMD monolayers on SiO_2_ substrates exhibit significant dielectric disorder that hinders exciton propagation, hBN-encapsulated samples offer a homogeneous dielectric background, where excitons can efficiently diffuse^1^. Another important aspect is the multi-valley band structure in TMDs. Electrons and holes can be located at different points of the Brillouin zone. This is especially relevant in tungsten-based TMDs, where the energetically lowest exciton states are known to be momentum-indirect or spin-forbidden transitions. Such excitons are effectively dark and therefore exhibit long lifetimes, enabling their long-range transport. Moreover, the coexistence of non-equilibrium populations in different valleys can result in intriguing transport phenomena, such as effective negative diffusion^2,3^.

A key parameter that governs exciton transport is the density of photo-excited electron-hole pairs. Exciton diffusion has been observed to slow down when going from low to intermediate densities—an effect that was attributed to the entropy ionization of bound excitons into an electron-hole plasma^4^. As the exciton density is increased and approaches 10^12^ cm^−2^, exciton–exciton interactions become relevant and can lead to an apparent increase of the effective diffusion coefficient. In particular, Auger scattering to high-energy states and the subsequent phonon emission cascade result in a strong temperature gradient of the exciton gas leading to an anomalous diffusion and even to the formation of excitonic spatial rings (halos)^5^. The carrier density can also be asymmetrically controlled with doping. By varying the doping level and measuring the diffusion coefficient of the overall electron-hole population, the interplay between trions, excitons, and free charge carriers becomes accessible. As trions acquire the oscillator strength of excitons and the charge of electrons (or holes), they can be in principle exploited as luminescent charge carriers.

While many fundamental aspects of exciton transport in monolayers have been unveiled in recent years, a comprehensive microscopic understanding of transport phenomena across a broad range of carrier densities has not been reached yet. In particular, the diffusion in the high-density regime at the Mott transition remains largely unexplored. Here, excitons become ionized as the inter-exciton distance approaches the exciton Bohr radius. At densities close to the Mott transition, the interplay and interactions between coexisting neutral and charged Coulomb complexes (such as excitons, trions, biexcitons, electron–hole plasma) is expected to play a crucial role. Moreover, Pauli’s exclusion principle should manifest at high carrier densities and low temperatures leading to the build-up of a Fermi pressure enhancing the diffusion^6^. The study of high-density transport is, however, very challenging. From the theoretical perspective, spatially and temporally resolved calculations of a system of coexisting and interacting charge complexes are very demanding. From an experimental point of view, it can be difficult to create such high carrier densities without damaging the sample. Moreover, a reliable analysis of experimental data becomes limited as it involves solving a non-linear drift–diffusion equation with too many open parameters. While the required advanced theoretical models and sophisticated experimental techniques for entering the high-density transport regime are certainly a demanding task, overcoming these challenges would enhance our understanding of exciton propagation and could open opportunities for novel exciton-based optoelectronic devices.

As the conventional control of transport properties via electric fields is not applicable for neutral excitons, manipulation via spatial energy gradients (induced e.g. by strain engineering) has been introduced in recent years. Inhomogeneous strain created e.g. by placing TMD monolayers on micropillars was shown to result in exciton funneling up to a micrometer range. Interestingly, in tungsten-based TMDs, an anti-funneling effect was demonstrated, i.e. a drift toward low-strain regions^7^. This unexpected behavior was suggested to be driven by specific momentum-dark excitons exhibiting a spectral blue-shift with increasing strain. However, the anti-funneling was observed in spatiotemperal PL spectra and the activation of these dark states at room temperature is still not well understood. Furthermore, faster and longer strain-induced funneling is desirable for technological applications. Hence, one of the key research goals is to realize longer exciton propagation, e.g. via steeper energy gradients. Moreover, in the context of technological application in exciton-based nanoelectronics, scalable strain engineering is required and could be reached via advanced nanoimprinting techniques^8^.

## Charge transfer excitons in lateral heterostructures

TMDs can form lateral heterostructures by growing two different monolayer materials during chemical vapor deposition resulting in a one-dimensional interface. The difference between band gaps and exciton energies at the two sides of the heterostructure leads to intriguing transport phenomena both across and along the interface. The energy offset gives rise to a diode-like unidirectional excitonic transport over a range of 400 nm toward the side with a lower energy^9^. Furthermore, these structures typically show a type-II band alignment, inducing spatially separated charge transfer excitons with potentially huge in-plane dipole moments^10^. The latter could induce fast high-density diffusion along the interface, with diffusion coefficients up to a few 100 cm^2^/s, which is three orders of magnitude larger than the corresponding exciton diffusion within the same material^11^. This is assumed to be a result of an interplay of dipole-dipole repulsion and Mott transition effects, resulting in excitonic-like highways allowing a long-range transport. However, the microscopic many-particle processes behind the diffusion across and along the interface in a lateral heterostructure have not been completely understood. In particular, the role of bound charge transfer excitons has remained unexplored. They could act as trapping states for propagating excitons and considerably affect the transport across the interface.

Lateral heterostructures are highly promising for optoelectronic devices, as the strong one-dimensional confinement leads to a remarkably fast exciton diffusion. Furthermore, charge transfer excitons can have significantly larger dipoles and smaller binding energies compared to interlayer excitons in vertical heterostructures. This facilitates an efficient charge separation and high controllability of the exciton transport with electrical fields. For technological applications, an improvement in the growth techniques is required to have scalable productions of lateral heterostructures with low disorder and small interface widths comparable with the excitonic Bohr radius.

## Interlayer excitons in vertical heterostructures

Van der Waals heterostructures consisting of vertically stacked TMD monolayers are regarded as a promising platform for realizing novel exciton-based devices and for investigating intriguing many-body correlations. In particular, they host spatially separated interlayer excitons which are long-lived and exhibit permanent out-of-plane dipole moments—properties which enable electrically tunable exciton transport in the micrometer range. Moreover, interlayer excitons exhibit strong repulsive dipolar interactions giving rise to a highly non-linear exciton propagation at elevated electron-hole densities^12^. Both electric fields and excitation power are crucial tuning knobs for engineering efficient long-range exciton transport. Despite some initial studies, the understanding of interlayer exciton propagation is still not fully revealed, especially in the high-density regime approaching the Mott transition. While the importance of the dipole-dipole repulsion has already been demonstrated, other non-linear processes, such as exciton-exciton annihilation or the interplay between excitons and higher-order charge complexes could strongly impact the exciton transport. One way of discriminating the impact of dipole-dipole repulsion from other interaction mechanisms is the variation of the layer separation between TMD layers, e.g. by including dielectric hBN spacers that can be tuned in thickness down to the monolayer hBN limit^12^.

Interestingly, the rich exciton landscape in van der Waals heterostructures does not only include intra- and interlayer excitons, but also layer-hybridized excitons, which carry both an intra- and an interlayer component. As such, these excitons possess both sizable oscillator strengths as well as out-of-plane dipole moments. It was recently demonstrated that electric fields can be used to tune the layer hybridization and thereby the size of the exciton dipole moment. In consequence, also the repulsive interaction between excitons can be electrically controlled^13^. Intriguingly, it is even possible to change the exciton ground state from a more interlayer-like state to a more intralayer state or vice versa, by applying out-of-plane electric fields. Recently, the large electrical tunability of TMD heterostructures has been exploited to demonstrate the efficient hybridization between bound and unbound exciton-hole complexes^14^.

Finally, the tunable exciton hybridization and many-body interactions in van der Waals heterostructures offer a promising tool to engineer exotic phases of matter, such as degenerate Bose-Fermi mixtures of excitons and electrons or exciton condensates as well as superfluidity. In particular, the intriguing interplay between bosons and fermions in Bose-Fermi mixtures could give rise to remarkable features including exciton-mediated superconductivity or supersolids/charge density waves depending on the nature and strength of the involved electronic and excitonic interactions^15^. By doping or optical injection of electron-hole pairs, the relative fermionic or excitonic occupations can be controlled, providing a precise way to tune these many-body interactions. Overall, strongly correlated phases have partially been experimentally realized for exciton-polaritons in 2D materials^16^, but finding unique experimental signatures and conclusive evidence of these phases of matter is highly challenging and remains an important venue of research.

## Moiré excitons in twisted heterostacks

When TMD monolayers are vertically stacked and twisted (or just being lattice-mismatched), a long-range moiré pattern is created, i.e. the local atomic configuration is changing periodically. Since electronic band gaps and wave function overlaps are strongly affected by the atomic registry, interlayer excitons as well as hybrid excitons become subject to a periodic superlattice potential. Recently, it was shown that the moiré potential in twisted MoSe_2_-WSe_2_ heterostructures significantly slows down the interlayer exciton diffusion at small twist angles^17,18^. The moiré potential traps excitons in low energy pockets impeding the free propagation. At increased twist angles, the moiré period becomes too short (compared to the Bohr radius) to confine excitons and they become mobile again. This has been further confirmed in spatiotemporal PL measurements revealing three distinct phases including trapped excitons at low exciton densities, weakly propagating excitons at intermediate densities, and mobile charges at densities larger than 10^12^ cm^−2^, where the Mott transition sets in and leads to an ionization of excitons^19^.

While the general impact of the moiré potential on the exciton transport has been observed, more advanced aspects of exciton propagation at elevated densities have still remained in the dark. In particular, non-linear transport phenomena, such as the interplay of excitons with higher-order charge complexes have not been thoroughly examined yet. Initial studies have investigated the electric field dependence of interlayer and hybrid excitons, but their twist-angle dependence and the impact of lattice reconstruction at small twist angles is missing. Furthermore, strain engineering has been performed on monolayers, but it remains challenging to deterministically create inhomogeneous strain profiles in twisted heterostructures.

Twisted heterostructures are extremely challenging both from the theoretical and experimental perspective. There is a large variety of exciton species that are differently affected by the stacking geometry and therefore see different moiré potential landscapes. Furthermore, interlayer interactions are manifold including stacking-dependent electrostatic shifts, interlayer-distance and valley-dependent tunneling, as well as deep strain-induced potentials arising from atomic reconstruction. The formation of moiré mini-bands with complex dispersion makes a semi-classical treatment of spatiotemporal exciton dynamics insufficient and quantum correlations play a key role in the case of flat bands. From the experimental perspective, it is highly challenging to control the twist angle and, so far, mostly small hand-made heterostacks have been fabricated containing many imperfections. Furthermore, spatial inhomogenities on length scales below optical resolution, unintentional strain, and irregular lattice reconstruction further worsen the reproducibility of transport studies. It is highly desirable to master these challenges, as twisted TMD heterostructures offer an unprecedented material class for exploring and designing quantum many-body states as well as for creating novel technological concepts. A large variety of possible material combinations and stackings, combined with externally accessible knobs, such as twist angle, strain, electric and magnetic fields, allow to create material platforms with tailored and largely tunable properties. In particular, the flat band physics occurring at low twist angles represents a widely unexplored field that is highly interesting both for fundamental science and technological applications.

## Conclusions

Transition metal dichalcogenides provide a material class with an exceptionally rich exciton landscape and intriguing transport phenomena that are technologically highly promising. The bottleneck towards exciton-based nanoelectronics is mastering challenges in the scalable production of high-quality samples of lateral and twisted vertical heterostructures as well as enabling microscopic modeling of interactions between coexisting neutral and charged Coulomb complexes that govern the propagation of excitons.
